# Supplementary material for: Inter-individual differences in pain anticipation and pain perception in migraine: Neural correlates of migraine frequency and cortisol-to-dehydroepiandrosterone sulfate (DHEA-S) ratio
Source: PLoS One. 2021 Dec 20;16(12):e0261570. doi: 10.1371/journal.pone.0261570 (PMC8687546; doi:10.1371/journal.pone.0261570)
Supplement: S1 Table — *p<0.05; 1point biserial correlation. (DOCX) [file pone.0261570.s001.docx]

**S1 Table. Spearman correlations of anxiety scores with age, education level and clinical characteristics (N=23).**

|  | trait anxiety | state anxiety | migraine frequency |
| --- | --- | --- | --- |
| state anxiety | 0.49* |  | -0.12 |
| age | -0.03 | 0.01 | 0.17 |
| education level^1^ | -0.12 | 0.12 | 0.01 |
| age at migraine onset | -0.10 | -0.05 | -0.19 |
| number of years with migraine | 0.14 | 0.15 | 0.25 |
| migraine frequency | -0.04 | -0.12 |  |

*p<0.05; ^1^point biserial correlation.
