# Supplementary material for: Inter-individual differences in pain anticipation and pain perception in migraine: Neural correlates of migraine frequency and cortisol-to-dehydroepiandrosterone sulfate (DHEA-S) ratio
Source: PLoS One. 2021 Dec 20;16(12):e0261570. doi: 10.1371/journal.pone.0261570 (PMC8687546; doi:10.1371/journal.pone.0261570)
Supplement: S2 Table — *p<0.05; 1point biserial correlation. (DOCX) [file pone.0261570.s002.docx]

**S2 Table. Spearman correlations of pre-scan raw cortisol, DHEAS and log cortisol-to-DHEAS with age, anxiety scores, clinical variables, menstrual phase and hormonal contraception taking (N=23).**

|  | pre-scan cortisol | pre-scan DHEAS | Log pre-scan cortisol-to-DHEAS ratio |
| --- | --- | --- | --- |
| BMI | -0.43* | 0.31 | -0.55* |
| trait anxiety | -0.15 | 0.11 | -0.20 |
| state anxiety | -0.25 | 0.08 | -0.23 |
| age | -0.12 | 0.16 | -0.23 |
| age at migraine onset | -0.10 | 0.30 | -0.26 |
| number of years with migraine | -0.11 | -0.16 | -0.01 |
| migraine frequency | 0.06 | 0.22 | -0.01 |
| menstrual phase^1^ (N=18) | 0.32 | -0.34 | 0.45 |
| hormonal contraception^1^ (N=18) | 0.05 | -0.30 | 0.30 |

*p<0.05; ^1^point biserial correlation. BMI: body mass index
