# Supplementary material for: Inter-individual differences in pain anticipation and pain perception in migraine: Neural correlates of migraine frequency and cortisol-to-dehydroepiandrosterone sulfate (DHEA-S) ratio
Source: PLoS One. 2021 Dec 20;16(12):e0261570. doi: 10.1371/journal.pone.0261570 (PMC8687546; doi:10.1371/journal.pone.0261570)
Supplement: S3 Table — Cluster-level familywise error rate of p<0.05; R, right; L, left; SMA, Supplementary motor area. (DOCX) [file pone.0261570.s003.docx]

**S3 Table. Activation changes during pain anticipation if state anxiety is controlled for (N=23).**

| Contrast | Cluster size (voxels) | Region | Br | Side | Peak T-value | MNI coordinates | | |
| --- | --- | --- | --- | --- | --- | --- | --- | --- |
|  |  |  |  |  |  | x | y | z |
| Pain cue – No pain cue | 237 | Cuneus |  | L | 5.58 | -6 | -91 | 14 |
|  |  | Lingual gyrus |  | L | 5.53 | -9 | -76 | -1 |
|  |  | Calcarine | 18 | R | 5.07 | 6 | -85 | 11 |
|  | 108 | Midcingulate |  | R | 4.48 | 12 | 14 | 41 |
|  |  | Midcingulate | 24 | L | 4.12 | -6 | 2 | 44 |
|  |  | SMA | 6 | R | 4.09 | 6 | 2 | 56 |
| No pain cue – Pain cue | 70 | Inferior occipital gyrus |  | L | 5.10 | -42 | -73 | -7 |
|  |  | Inferior occipital gyrus |  | L | 4.96 | -42 | -61 | -13 |
|  | 70 | Superior parietal lobule |  | R | 5.04 | 33 | -61 | 53 |
|  |  | Superior occipital gyrus |  | R | 4.60 | 27 | -61 | 32 |

Cluster-level familywise error rate of p<0.05; R, right; L, left; SMA, Supplementary motor area.
