# Supplementary material for: Inter-individual differences in pain anticipation and pain perception in migraine: Neural correlates of migraine frequency and cortisol-to-dehydroepiandrosterone sulfate (DHEA-S) ratio
Source: PLoS One. 2021 Dec 20;16(12):e0261570. doi: 10.1371/journal.pone.0261570 (PMC8687546; doi:10.1371/journal.pone.0261570)
Supplement: S7 Table — Cluster-level familywise error rate of p<0.05; R, right; L, left. (DOCX) [file pone.0261570.s007.docx]

**S7 Table. Activation changes to painful and non-painful stimulation if gender was controlled for (N=23).**

| Contrast | Cluster size (voxels) | Region | Side | Peak T-value | MNI coordinates | | |
| --- | --- | --- | --- | --- | --- | --- | --- |
|  |  |  |  |  | x | y | z |
| Pain – No pain | 1138 | Vermis 8 | L | 7.58 | -3 | -64 | -28 |
|  |  | Cerebellum_6 | R | 5.90 | 33 | -49 | -31 |
|  |  | Cerebellum_6 | R | 5.89 | 33 | -55 | -25 |
|  | 199 | Postcentral gyrus | L | 5.76 | -57 | -19 | 17 |
|  |  | Supramarginal gyrus | L | 5.46 | -63 | -28 | 29 |
|  |  | Insula | L | 4.89 | -39 | -16 | 8 |
|  | 354 | Heschl gyrus | R | 5.55 | 42 | -22 | 11 |
|  |  | Middle temporal gyrus | R | 5.42 | 45 | -43 | -1 |
|  |  | Rolandic operculum | R | 4.96 | 60 | -1 | 5 |

Cluster-level familywise error rate of p<0.05; R, right; L, left.
